# Study of the Effect of Inorganic Particles on the Gas Transport Properties of Glassy Polyimides for Selective CO_2_ and H_2_O Separation

**DOI:** 10.3390/membranes8040128

**Published:** 2018-12-09

**Authors:** Sara Escorihuela, Lucía Valero, Alberto Tena, Sergey Shishatskiy, Sonia Escolástico, Torsten Brinkmann, Jose Manuel Serra

**Affiliations:** 1 Instituto de Tecnología Química, UniversitatPolitècnica de València-Consejo Superior de Investigaciones Científicas, Avda. Los Naranjos, s/n 46022 Valencia, Spain; saesro@itq.upv.es (S.E.); valeroromerolucia@gmail.com (L.V.); soesro@upvnet.upv.es (S.E.); 2 Helmholtz-ZentrumGeesthacht, Institute of Polymer Research, Max-Planck-Str.1, 21502 Geesthacht, Germany; torsten.brinkmann@hzg.de

**Keywords:** mixed matrix membranes, carbon dioxide, water vapor permeability, polyimides, inorganic fillers, gas separation membranes, water transport

## Abstract

Three polyimides and six inorganic fillers in a form of nanometer-sized particles were studied as thick film solution cast mixed matrix membranes (MMMs) for the transport of CO_2_, CH_4_, and H_2_O. Gas transport properties and electron microscopy images indicate good polymer-filler compatibility for all membranes. The only filler type thatdemonstrated good distribution throughout the membrane thickness at 10 wt.% loading was BaCe_0.2_Zr_0.7_Y_0.1_O_3_ (BCZY). The influence of this filler on MMM gas transport properties was studied in detail for 6FDA-6FpDA in a filler content range from one to 20 wt.% and for Matrimid^®^ and P84^®^ at 10 wt.% loading. The most promising result was obtained for Matrimid^®^—10 wt.% BCZY MMM, which showed improvement in CO_2_ and H_2_O permeabilities accompanied by increased CO_2_/CH_4_ selectivity and high water selective membrane at elevated temperatures without H_2_O/permanent gas selectivity loss.

## 1. Introduction

Mixed matrix membranes (MMMs) [1,2] are considered as a very promising route to overcome limitations of the Robeson upper bound of the permeability/selectivity relationship by combining good mechanical, but rather disappointing gas transport properties of polymers, with excellent diffusion and sorption properties of inorganic porous media having very poor mechanical properties, e.g., flexibility [3].

Gas separation membranes have been on the market since 1980 [4] and have proven their reliability [5]. Unfortunately, since the boom of membranes introduction into the market at the end of the 20th century, not too many new membranes reached practical application level for gas separation processes. The reason for it lays in the versatility of existing membrane technology that allows the combination of membrane separation stages with other unit operations and to achieve goals of the separation process [6]. New membranes introduced to the market should exhibit significantly better properties, as compared to those already commercially available. At this point, the combination of properties of polymers and inorganic substances able to selectively transport gas or vapor molecules becomes very appealing [7].

The requirements for polymers to be used in membranes are: Adequate gas transport properties (balance between permeability and selectivity), processability as a thin film, and high reproducible manufacturing from batch to batch. For the formation of MMMs, good adhesion between the polymer matrix and the inorganic filler is essential, especially in case of polymers with high *T_g_* (glass transition temperature) [8], good mechanical properties and stability of properties as a function of time.

The inorganic fillers to be used in MMMs should have (i) particles as small as possible since selective layers of modern polymer based gas separation membranes have a thickness of 100 nm and less; (ii) good affinity to the polymer; and (iii) gas transport properties matching, for the target gas in the separation process, those of the matrix polymer [9]. Several fillers have been studied in order to be combined with a polymer matrix, i.e., zeolites, carbon mesoporous silica, and metal organic frameworks (MOF) [10,11,12,13,14,15]. Regarding this last particular example, MOFs appear to be especially attractive, as concerns compatibility with polymers [16]. However, rather few reported works on MMFs with MOFs evidence an enhancement of CO_2_ permeability and CO_2_/CH_4_ selectivity, as compared to the pure polymer membrane [17].

**Figure 1:**
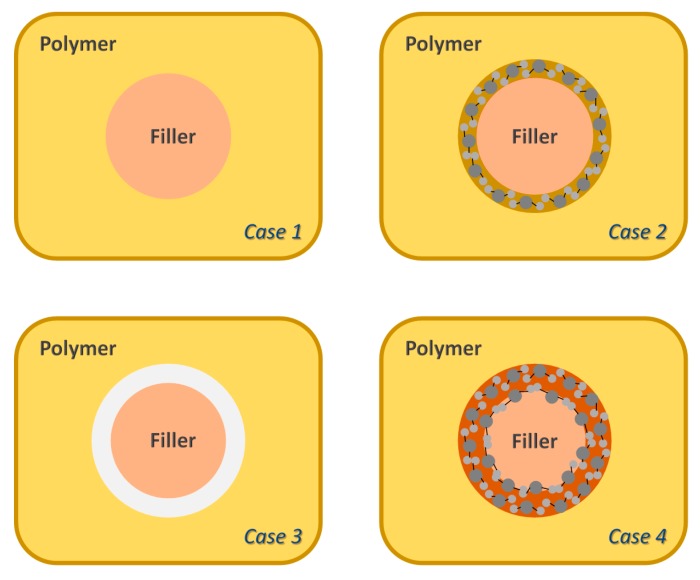
Schematic diagram of various structures for MMMs [38,39].

Depending on the compatibility of the filler and the polymer matrix, four different cases (Figure 1) and the expected effect on the separation properties can be described [18,19,20,21,22]. Case 1 is an ideal situation, where the filler is perfectly incorporated into the polymer matrix. This situation can result in an improvement of the separation properties of the mixed matrix material. In Case 2, there is a rigidification of the polymer matrix in the area around the filler. This normally results in an increase of the selectivity, due to the increased rigidity, but also in a decrease of the permeability. This is normally confirmed by an increase on the *T_g_* of the MMMs. Case 3 exhibits a creation of an interphase due to the incompatibility between the particle and the polymer matrix. This results not only in an increase of the permeability due to the bigger fractional free volume, but also in a decrease of the selectivity. In Case 4, the polymer matrix penetrates the pore or free volume of the filler. In this case, both permeability and selectivity decrease.

In this work, several MMMs were studied for gas transport properties with focus on CO_2_, CH_4_ and H_2_O as components in various industrially relevant gas mixtures, for example, natural gas, biogas as well as gas streams in chemical and petrochemical industries [23,24,25,26]. Biogas and natural gas are considered as the most environmentally friendly resources for large scale electric energy production and as sources with significant methane content, which is involved in several relevant reactions such as combustion, steam reforming, or halogenation. For example, in Germany, natural gas contains about 95% CH_4_ and not more than 2% CO_2_ concentration in the gas pipelines [27,28,29]. On the other hand, biogas is a mixture of several gases produced by the anaerobic decomposition of organic matter. Biogas mainly consists of methane (50–70%), carbon dioxide (30–50%), and other compounds including hydrogen sulfide (H_2_S), water, and other trace gas compounds [30]. The membranes considered in this study could be used to separate CO_2_ from these gases. Additionally, these membranes could also be applied to remove water at low temperature in several combustion processes in order to recover it and on-site reuse for other purposes.

In the current study, three polyimides and six inorganic fillers were used to prepare MMMs as thick films and the corresponding gas transport properties for CO_2_, CH_4_, and H_2_O were systematically studied. Polyimides were selected due to their outstanding gas transport properties (low permeability coefficients, high selectivity, and good thermal stability) for several gas pairs as CO_2_/CH_4_ [31] or O_2_/N_2_ [32], and in case of the studied polymers, excellent film forming properties. The six inorganic materials used as nano-sized particles were selected, taking into account the expected good affinity for gas molecules as CO_2_ and water vapor [33]. BCZY, LaWO, 8YSZ, and La_2_O_3_ were selected for the CO_2_ and H_2_O affinity due to their basicity and/or the important water absorption that present [34,35,36], whereas zeolites have been previously used as fillers in MMM for high-temperature CO_2_ separation [37].

## 2. Experimental Section

### 2.1. Materials

#### 2.1.1. Polymers

Three different polyimides were employed in the present study: 6FDA-6FpDA, Matrimid^®^ 5218, and P84^®^. The 6FDA-6FpDA polyimide was synthesized following the classical in-situ silylation two steps method [40]. A detailed description of this synthesis can be found in a previous work [41]. Polyimides P84^®^ and Matrimid^®^ 5218 were purchased from HP Polymer GmbH (Lenzing, Austria) and Huntsman Advanced Materials (Salt Lake City, UT, USA), respectively.

#### 2.1.2. Solvents

Tetrahydrofuran (THF), N-methyl-2-pyrrolidon (NMP), dimethylformamide (DMF), dimethylacetamid (DMAc), toluene, chloroform, and isopropanol for analysis grade were purchased from Merck (Darmstadt, Germany) and used as received.

#### 2.1.3. Particles

Six inorganic fillers were employed: 8 mol.% Yttria Stabilized Zirconia (8YSZ), La_2_O_3_, La_5.4_WO_12_ (LaWO), BaCe_0.2_Zr_0.7_Y_0.1_O_3_ (BCZY) and two zeolites (ITQ-2 and Beta). 8YSZ powder was provided by Tosoh Corporation (Tokyo, Japan). La_2_O_3_ was synthesized by co-precipitation from lanthanum nitrate (La(NO_3_)_3_) and subsequent calcination at 800 °C for five h. LaWO, provided by CerPoTech (Tiller, Norway) in powder form was calcined at 800 °C for six h. BCZY powder, also provided by CerPoTech, was calcined at 950 °C for sixh. Nanocrystalline Beta zeolite (BEA material) and ITQ-2 (delaminated MCM-22 zeolite material) [42] were synthesized by the ITQ (Instituto de Tecnología Química, Valencia, Spain) and are here used after calcination (organics removal) in its acidic form. All the fillers were ball-milled previously for 24 h. Table 1 shows a summary of the fillers properties.

### 2.2. Membranes Fabrication

#### 2.2.1. Inorganic Particles Dispersion

Different solvents such as THF, NMP, DMF, DMAc, toluene, chloroform, and isopropanol were tested for the dispersion of the particles. The ultrasonic devices used to disperse the fillers were the digital Sonifier^®^, models 250 and 450 (BRANSON Ultrasonics Corporation, Danbury, CT, USA). The dispersion was carried out with the pulse/pause mode, in particular, pulse off for one second and pulse on for one second for a total duration of 30 min. In addition, the dissolution container was inside an ice bath to avoid the sample heating during the dispersion process. In all the cases, the best dispersions and the most visually stable over time (smallest degree of sedimentation) were obtained by using NMP. The zeolites were fully suspended, whereas in the dispersions obtained with 8YSZ, La_2_O_3_, LaWO, and BCZY with some sedimentation with time observed.

#### 2.2.2. Membranes Formation

Mix matrix membranes (MMMs) were made with 250 mg of polymer and inorganic fillers and 2.25 g of solvent. First, the inorganic fillers were dried at 120 °C for 24 h before the membrane preparation. Then, the fillers were dispersed in 1 g of NMP, as a solvent, by using an ultrasound device. At the same time, the polymer was dissolved with the rest of the solvent. The particle suspension was finally added to the polymer solution, obtaining a homogeneous solution of polymer and fillers. Subsequently, membranes were prepared by following the solvent evaporation method. The mixed matrix solutions were poured into metal rings placed on a heating plate at 70 °C for 12 h. Then, the membranes were heat treated following the steps: (a) 100 °C under vacuum for 1.5 h, (b) 200 °C under vacuum for 2 h, and (c) cooling to room temperature under vacuum.

### 2.3. Samples Characterization

Thermogravimetric analysis (TGA) experiments were carried out on the thermal analysis instrument NETZSCH TG 209 F1 Iris (Netzsch GmbH, Selb, Germany) in order to evaluate the thermal stability of the MMM and quantify the percentage of fillers. Disc samples with weights of between five and 15 mg were cut from the pieces obtained as described in Section 2.2. The TGA experiments consisted of two steps: (i) First, the sample was heated from 30 °C to 800 °C at 10 °C/min under an argon flow (dynamic scan); and (ii) once 800 °C was reached, the temperature was maintained for 30 min under synthetic air (static scan), in order to burn out the organic from the samples. The precision for the weight determination is ±0.1 μg. Differential scanning calorimetry (DSC) analysis was used to determine the *T_g_* of polymers. DCS experiments were carried out with a calorimeter DSC 1 (Mettler Toledo, Columbus, OH, USA) at a heating rate of 10 K/min under nitrogen atmosphere to prevent oxidation. The glass transition is determined, with a precision of ±0.2 K, in the second heating cycle to avoid the history effect of sample.

The apparent molecular weight of the polymers and the MMMs was determined by Gel permeation Chromatography (GPC) after calibration with polystyrene standards. GPC measurements were performed at 40 °C having DMAc as eluent on a Waters instrument (Waters GmbH, Eschborn, Germany) equipped with polystyrene gel columns of different pore sizes, using a refractive index (RI) detector.

The XRD analysis were performed by using a D8 DISCOVER X-ray diffractometer (Bruker, Billerica, MA, USA). The range of measured Bragg angles was from 2 to 82°, with an increase of 10°. A 50 kV voltage and 1000 μA current was used.

The morphology of prepared MMMs and particle distribution throughout the membrane cross-section were analyzed using a scanning electron microscope (SEM) “Merlin” (Zeiss, Oberkochen, Germany). Samples for cross-sectional images were prepared by breaking the membrane immersed into liquid nitrogen and subsequent coating with a 4 nm carbon layer.

The permeability, diffusivity and solubility coefficients of CH_4_, CO_2_ and H_2_O vapor in the manufactured membranes were determined by using the well-known constant volume, variable pressure method, i.e., the “time-lag method” [40]. The basic principle is the measurement of the transitory response at the downstream part of a membrane to a pressure step at the upstream part, that is, the time-lag (*t*_0_), which is graphically determined as the intersection of the line drawn through the linear region of the pressure increase curve to intersection with the time axis [49]. The diffusion coefficient (*D*) is linked to the time-lag (*t*_0_) through the Equation (1).

(1) $$t_{0}=\frac{l^{2}}{6\cdot D}$$

where *l* is the thickness of the membrane. The permeability coefficient (*P*) can be obtained from the range where the permeate pressure increases linearly (Equation (2)).

(2) $$P=D\cdot S=\frac{V_{p}l\left(p_{p2}-p_{p1}\right)}{ART\Delta t(p_{f}-\left(p_{p2}+p_{p1}\right))}$$

where *V_p_* is the constant permeate volume, *l* is the film thickness, *A* is the effective area of the membrane, *R* is the gas constant, ∆*t* is the time of the permeate pressure increase from *p*_*p*1_ to *p*_*p*2_, and *p_f_* is the feed pressure. Finally, solubility coefficient (*S*) can be obtained with the permeability and the diffusion coefficient by means of Equation (3).

(3) $$S=\frac{P}{D}$$

The measurements were made at different temperatures and at one bar of feed pressure. For each gas measurement, the facility was evacuated until no desorption from the membrane was observed and the gas to be measured was subsequently refilled. The feed and permeate sides of the membrane are connected to a vacuum pump with valves and additional valves that connect the feed side with several gases.

Experiments on water vapor transport were carried out as follows: The pressure vessel keeping feed pressure of a gas or vapor under study at a constant level during the acquisition of the time-lag curve was filled with water vapor corresponding to the saturation pressure at a given temperature. For the beginning of the experiment, the feed pressure vessel was connected to the previously evacuated measurement cell by opening vacuum valves. It caused a drop of the vapor pressure to approximately 70% of vapor activity at a given temperature. Due to the design of the vacuum system of the “time-lag” facility, it is not possible to carry out vapor measurements at activities higher than 70%. After the time-lag curve was recorded the system was completely evacuated and experiment repeated for 3 times. For the CH_4_ and CO_2_, gas properties were studied at temperatures up to 80 °C.

## 3. Results and Discussion

### 3.1. Thermal Properties

**Figure 2:**
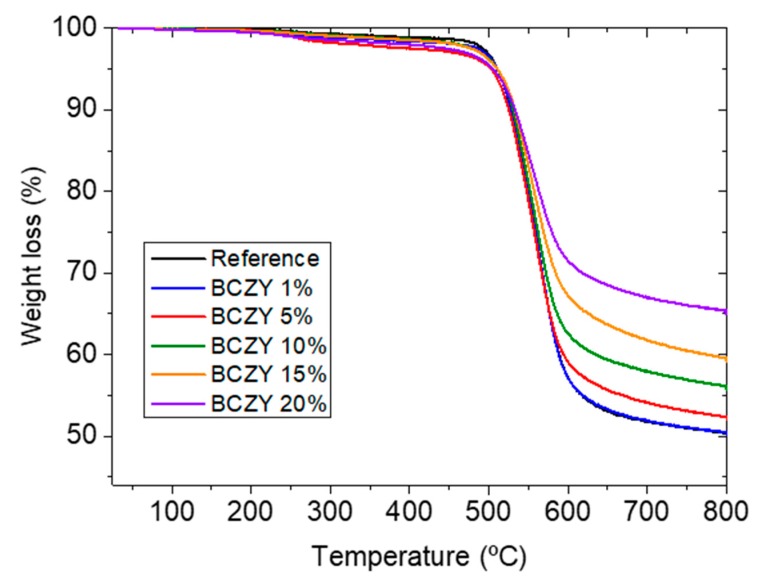
TGA graphs for the results of 6FDA-6FpDA with different percentage of the BCZY particles.

Thermogravimetric analysis experiments (TGA) were performed in order to evaluate the thermal stability of the MMMs and to additionally determine the real amount of particles in the final sample [50]. The parameter used to evaluate the quantity of particles in the membranes is the residual mass (*R_M_*). In Table 2, the TGA results for three different cases studied in this work are represented: 6FDA-6FpDA with 10 wt.% of the six different particles, 6FDA-6FpDA with different percentage of the BCZY particles, and 6FDA-6FpDA, Matrimid^®^ and P84^®^ with 10 wt.% of BCZY. It can be appreciated that the temperature of the maximum weight loss (*T_max loss_*) is a characteristic property of the polymer matrix, i.e., 550 °C for 6FDA-6FpDA, 560 °C for Matrimid^®^, and finally, 580 °C for P84^®^. The value of the residual mass *R_M_*, determined after exposure of the sample to synthetic air at 800 °C, indicates the content of inorganic particles within the sample. The low deviation from the theoretical values observed for 10 wt.% 8YSZ, La_2_O_3_, LaWO, and BCZY in 6FDA-6FpDA indicates a good dispersion and adhesion of these particles in the polymer matrix [37,51]. On the contrary, in the case of the zeolites (ITQ-2 and Beta), an important difference between nominal and experimental content occurs. This difference is attributed to the nanometer size of the particles that may cause a loss of part of the constituting elements of these particles. On the other hand, 6FDA-6FpDA + 20 wt.% BCZY and Matrimid^®^ + 10 wt.% BCZY present an *R_M_* value higher than the initial percentage of particles that indicates an irregular distribution of the polymer chains around the particles. Additionally, an identical temperature of the maximum weight loss *T_max loss_* for 6FDA-6FpDA with different percentages of the BCZY particles is observed in Figure 2. From these results, it can be concluded that the incorporation of inorganic particles to the polymer matrix does not affect the thermal stability of the polymer.

The glass transition temperature (*T_g_*) of these three polymers was determined by differential scanning calorimetry (DSC). An evolution of the *T_g_* with the rigidity of the polymer chains following the order of rigidity 6FDA-6FpDA < Matrimid^®^ < P84^®^ was found. For all the 6FDA-6FpDA-based MMMs, the *T_g_* decreases in comparison to the pure polyimide, except for the BCZY membrane that presents a *T_g_* similar to the reference. The observed reduction of the *T_g_* may indicate a plasticization effect introduced by the filler particles.

When the behavior of the *T_g_* for different BCZY contents in the polymer matrix was analyzed, only small irregular changes were found, i.e., an increase of *T_g_* for low BCZY percentages between one and 5 wt.% and a similar value for 10 wt.%, which may indicate a small macromolecular chain rigidification [52] (see Table 2). This could have an effect on the gas transport properties, expecting an increase in selectivity but decrease of the permeability coefficients.

### 3.2. Microstructure Characterization

**Figure 3:**
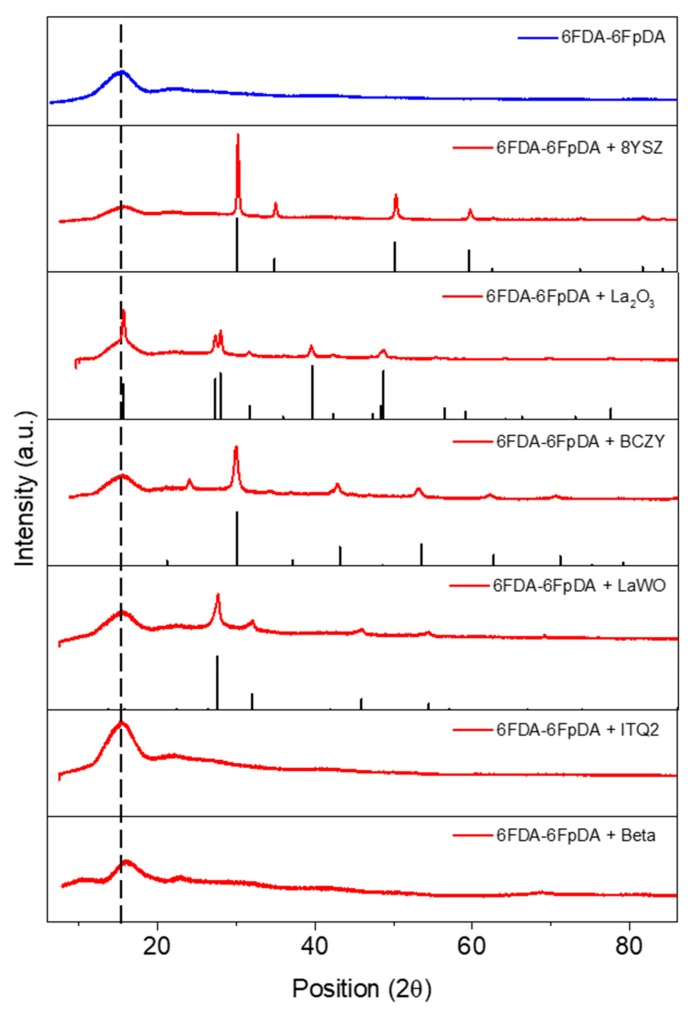
X-ray diffraction patterns for 6FDA-6FpDA with 10 wt.% fillers (red lines) and reference patterns corresponding to 8YSZ, La(OH)_3_, LaWO, and BCZY crystals (black peaks).

The X-ray diffraction (XRD) technique is used to evaluate the interaction between the selected particles with the polymer matrix. Figure 2 shows the X-ray spectra of the MMMs made of 6FDA-6FpDA with 10 wt.% of particles and the reference patterns of 8YSZ, La(OH)_3_, LaWO, and BCZY compounds. Regarding La_2_O_3_, this inorganic filler is highly hygroscopic, which may affect its crystalline structure. Actually, this was the case in this work, and as revealed in Figure 3, the pattern of La_2_O_3_ changed to La(OH)_3_. XRD patterns demonstrate that the particles are well integrated in the polymer matrix, showing the combination of the diffraction pattern of the polymer and particles. Nevertheless, in the case of 8YSZ, La_2_O_3_, and Beta zeolite, the main peak of the polymer matrix moves to higher values of 2*θ*, and therefore, the polymer intersegmental distance decreases [53,54,55]. This may indicate that part of the fraction free volume (FFV) of the polymer can be altered in the vicinity to the embedded particle.

**Figure 4:**
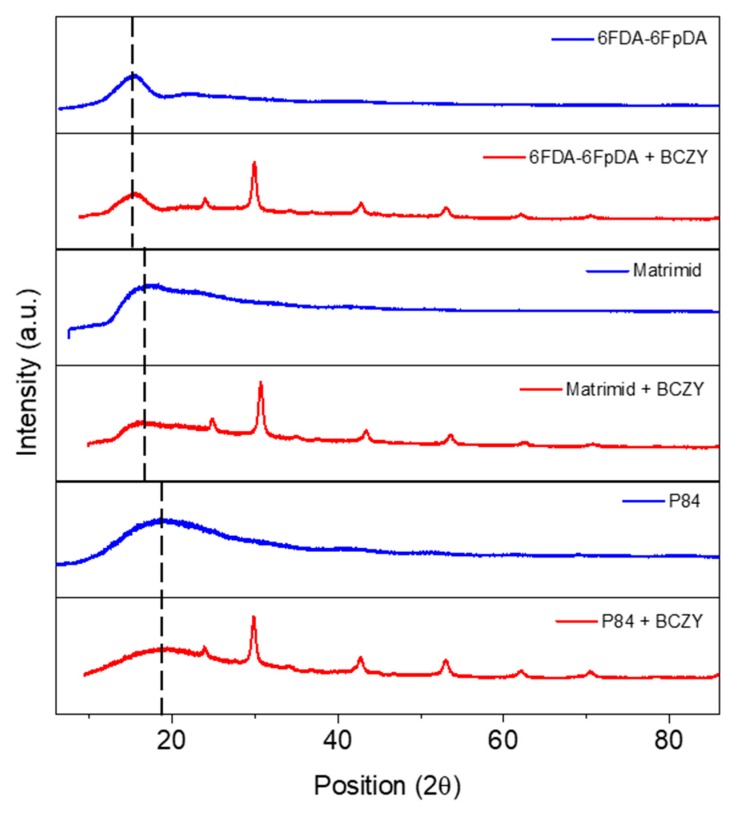
X-ray patterns for different polymer matrix:Pure, blue lines, and with 10 wt.% BCZY, red lines.

Figure 4 displays the X-ray diffraction pattern of MMMs made of 6FDA-6FpDA, Matrimid^®^ and P84^®^ combined with BCZY 10 wt.% and illustrates how the intersegmental distance between polymer backbones are directly related to the FFV (6FDA-6FpDA > Matrimid^®^ > P84^®^) [56,57].

The fillers distribution, sedimentation, and agglomeration in the membranes were analyzed based on scanning electron microscope (SEM) cross-sectional images.

**Figure 5:**
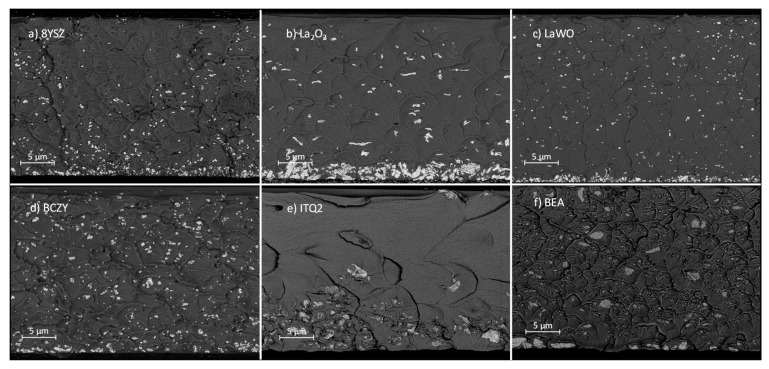
SEM images (fracture cross-sections) for 6FDA-6FpDA MMM and 10 wt.% fillers.

The cross-section of 6FDA-6FpDA MMMs with 10 wt.% fillers (Figure 5) shows that part of the inorganic fillers forms agglomerates. Theoretically, the size of particles (Table 1) should be in the nanometer range, but most of the fillers occur as agglomerates of embedded single nanoparticles. Additionally, sedimentation of particles can also be appreciated, i.e., there is a certain sedimentation for most fillers. These two phenomena may be associated with the dynamics of the membrane formation process. In general, the process for solvent evaporation is slow, and it gives enough time for particles for sedimentation to the bottom, which is something very common in thick film MMMs. This is related to the solvent used, NMP, that exhibits a high boiling point and to the high density of most of the studied filler particles (Table 1). When all the inorganic fillers are compared, it is ascertained that La_2_O_3_ showed the largest amount of agglomerates, and in general, a poor distribution. In contrast, BCZY is the filler with the best particle distribution throughout the membrane thickness, and the least agglomeration and sedimentation.

### 3.3. Gas Transport Properties

Gas transport properties for the three different cases (i.e., particle type, particle content and polymer type) were evaluated. Time lag equipment was used to study, not only the permeability and the selectivity, but also the solubility and the diffusivity coefficients of gases and water vapor in MMMs as a function of temperature.

#### 3.3.1. Influence of the Particle Type

In this section, MMMs composed by 90 wt.% of 6FDA-6FpDA as polymer matrix and 10 wt.% of different fillers are characterized Table 3 shows how the particles influence the polymer matrix reference (6FDA-6FpDA) in terms of permeability and selectivity at 30 °C. Permeability variations are always negative, and on the contrary, the selectivity variations are positive in all the cases. The decrease in permeability with the addition of inorganic fillers can be related to the formation of a densified layer of polymer on the polymer/particle interphase (case 2), although a reduction of the *T_g_* was not observed (Table 2). Polymer densification leads to reduced free volume, and consequently, to higher selectivity for the gas pair with significantly different kinetic diameters of gas molecules. Interestingly, the decrease in CO_2_ permeability leads to significant, reverse-proportional increase of the activation energy of CO_2_ permeability [58]. This observation allows one to conclude good contact between polymer and inorganic particle, i.e., presence of no gaps in this interface [18,59].

**Figure 6:**
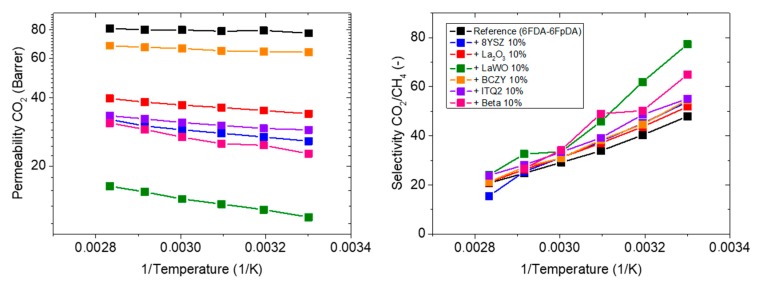
Permeability of CO_2_ and CO_2_/CH_4_ selectivity for the MMM composed by 90 wt.% of 6FDA-6FpDA and 10 wt.% of different fillers as a function of temperature.

The permeability of CO_2_ and selectivity for the gas pair CO_2_/CH_4_ was also measured as a function of temperature. CO_2_ permeability increases with temperature for all tested MMMs. However, none of them exhibits higher permeability values than the reference membrane, as is observed in Figure 6 (left-hand). Selectivity decreases as a function of temperature, while the effect of the type of filler becomes more visible and relevant at lower temperatures, because the differences between them are more evident. Table 3 displays the activation energy for the different fillers and the reference. The activation energy of the MMMs is higher than the activation energy of the polymeric membrane 6FDA-6FpDA. Hence, the formation of a rigid layer around the particles is confirmed.

**Figure 7:**
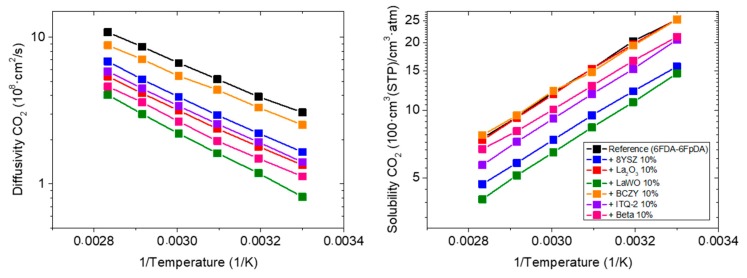
Diffusivity and solubility coefficient of MMMs composed by 90 wt.% of 6FDA-6FpDA and 10 wt.% of different fillers, as a function of temperature.

Taking into consideration all the MMMs, the sample with BCZY particle exhibits the highest permeability value, as well as a notable increase in CO_2_/CH_4_ selectivity. All MMMs exhibit worse permeability than the reference, while a visible improvement in selectivity can be ascertained. Gas permeability through a membrane depends on two parameters, diffusivity coefficient and solubility coefficient (Figure 7). The solubility coefficient was not improved by the incorporation of particles. In particular, the membranes containing BCZY and La_2_O_3_ particles have practically the same CO_2_ solubility coefficient as the pure polymer, but the rest of inorganic fillers causes a CO_2_ solubility coefficient decrease, and all of them decrease as a function of temperature.

On the contrary, the diffusivity coefficient, which is mainly determined by the FFV, increases as a function of temperature for all the MMMs. It is assumed that particles are blocking part of the FFV of the polymer matrix, and hence, the final permeability decreases. Observing the evolution of the diffusivity coefficient with temperature, this statement can be confirmed, since the reference diffusivity coefficient is higher than the rest at room temperature, and this difference is bigger at higher temperatures.

As a conclusion, BCZY, among all the studied fillers, was the most promising particle for MMM due to the fact that it is the particle that exhibits the highest permeability value, as well as a notable increase in CO_2_/CH_4_ selectivity. Therefore, this nano-sized filler was selected for the next step, i.e., evaluation of the influence of the amount of nanoparticles on the separation performance.

#### 3.3.2. Influence of the Particle Content

In order to understand the influence of the inorganic filler concentration on MMM transport properties, membranes were prepared with different BCZY content in weight percent 1%, 5%, 10%, 15% and 20% in the same polymer matrix, i.e., 6FDA-6FpDA. Table 4 summarizes permeability and ideal selectivity data obtained for the aforementioned membranes at 30 °C.

As in the previous section, the permeability values for the different filler concentration are lower than the permeability of the reference membrane (6FDA-6FpDA). In contrast, regarding the selectivity variation, the MMM with the highest content of particles has a negative effect on selectivity. This suggests that not only the filler particles are blocking the FFV—decreasing the permeability—but also aggregates influence the selectivity negatively.

Permeability of CO_2_ and selectivity of the gas pair CO_2_/CH_4_ was measured as a function of temperature. Activation energies for the MMMs of 6FDA-6FpDA with different % of BCZY filler are indicated in Table 4. Permeability of CO_2_ increases with temperature but decreases with the content of BCZY fillers. All the membranes with BCZY exhibit higher permeability values than with the other fillers studied in the previous section (see Figure 6). This fact indicates that BCZY is the most promising particle. Regarding the selectivity of CO_2_/CH_4_, it decreases as a function of temperature, and there is not a wide difference between the reference polymer and MMMs. No clear dependence between BCZY content and activation energy can be observed. Therefore, in the next sub-section, the filler BCZY at 10 wt.% is tested for two other polymers that present lower FFV, that is, with lower permeability, in order to check if the addition of particles enables it to improve their separation performance.

#### 3.3.3. Influence of the Polymer Matrix

The influence of different polymeric matrices with different FFV (6FDA-6FpDA, Matrimid^®^ and P84^®^) on the separation properties is studied, employing the results of the previous studies, that is, the most suitable filler (BCZY) and the most suitable proportion (10 wt.%) [60]. Table 5 shows the polymer matrix influence (6FDA-6FpDA, Matrimid^®^ and P84^®^) in terms of permeability and selectivity at 30 °C. We hypothesized that, for lower permeable polymers, the particles may positively affect the gas separation properties of the mixed matrix membranes [61,62,63]. As is shown in the table below, the Matrimid^®^ mixed matrix membrane exhibits higher permeability, as well as selectivity, compared to its reference (case 1 in Figure 1), while the P84^®^ mixed matrix membrane behaves similar to the 6FDA-6FpDA mixed matrix membrane, with a decrease in permeability and an increase in selectivity (case 2 in Figure 1) [59].

**Figure 8:**
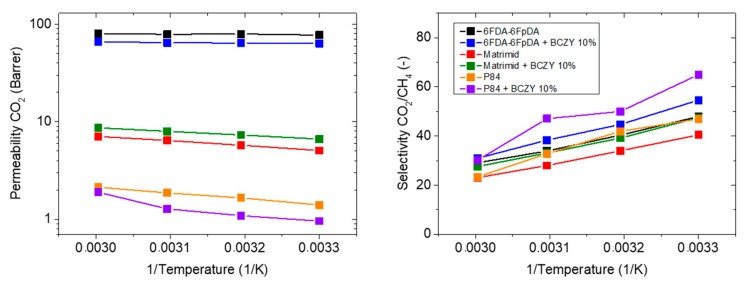
Permeability of CO_2_ and CO_2_/CH_4_ selectivity for the membranes made of 6FDA-6FpDA, Matrimid^®^ and P84^®^ with and without 10 wt.% BCZY as a function of temperature.

The permeability of CO_2_ and the selectivity of the CO_2_/CH_4_ gas pair were determined as a function of temperature, see Figure 8. All polymers show good compatibility with BCZY particles, but behave differently, as casted MMMs. 6FDA-6FpDA and P84^®^ show lower permeability coefficients of CO_2_ in MMM form while Matrimid^®^ with 10 wt.% of BCZY shows an improvement in both CO_2_ permeability and CO_2_/CH_4_ selectivity. As it can be expected for the CO_2_/CH_4_ gas pair, the ideal selectivity decreases with temperature for both pure polymer and MMMs.

**Figure 9:**
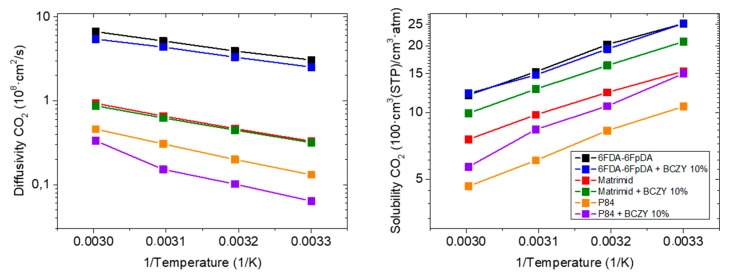
Diffusivity and solubility coefficient of CO_2_ of membranes made of 6FDA-6FpDA, Matrimid^®^ and P84^®^ with and without 10 wt.% BCZY as a function of temperature.

Figure 9 shows diffusion and solubility coefficients of CO_2_ as a function of temperature. It can be ascertained that the permeability of Matrimid^®^ with BCZY particles is higher than the pure polymer Matrimid^®^ because solubility coefficients improve, and diffusivity coefficients remain constant. In general, the activation energy of CO_2_ diffusivity (Figure 9) of the polymer membrane is not influenced by the incorporation of BCZY particles suggesting that the diffusion mechanism is apparently not affected.

#### 3.3.4. Transport of Water Vapor in MMMs

All the MMMs tested until now exhibits high CO_2_ solubility coefficients, which may be related to the water vapor transport properties. Additionally, the fillers selected for this work exhibit a high affinity towards water. They can be perspective materials for membranes able to remove water from CO_2_-rich gas streams as combustion tail-gas or catalytic converters products. Hence, all prepared membranes were tested for water vapor transport using the “time-lag” setup.

Table 6 summarizes water vapor permeability’s and the activation energies for all the MMMs studied in this work. The achieved permeability values are remarkably high, although permeability decreases as a function of temperature. The negative values of the activation energy are related to the exothermic nature of the water solubility and its effect dominates in the overall separation process. In all cases, water permeability decreased in MMMs compared to pure polymers except the case of Matrimid^®^ with 10 wt.% of BCZY. This filler showed the highest permeability coefficient of MMMs when compared to other fillers mixed with 6FDA-6FpDA.Looking at activation energies of water permeability in 10 wt.% MMMs based on three polymers, one may conclude that the “slower” polymers Matrimid^®^ and P84^®^ are more benefitting by the incorporation of BCZY. Namely, P84^®^-based MMM shows slightly positive E_a_ (P) for water vaporand Matrimid^®^ MMM exhibits a higher water permeability coefficient than for the pure polymer and a lower corresponding activation energy. These two materials are appealing materials for further application in membrane-assisted water vapor removal from combustion streams or in catalytic reactors in order to shift chemical equilibrium in CO_2_-hydrogenation reactions.

## 4. Conclusions

A complete study of the influence of inorganic fillers on the gas transport properties of different polyimides, typically applied in gas separation processes, i.e., purification of biogas and natural gas was developed. The strategy of this work was the incorporation of inorganic nanoparticles as MMM fillers. These inorganic materials were selected because the expected good affinity for gas molecules as CO_2_ and water vapor. The particles were successfully dispersed and incorporated into the polymer matrix. Consequently, 15 different MMMs were produced as a thick film. Materials were characterized by different techniques including TGA, XRD, DSC, or SEM. Gas transport properties were evaluated for CH_4_, CO_2_, and H_2_O at temperatures from 30 to 80 °C in a time lag equipment.

It was observed that, in general, the inorganic fillers could produce small rigidification in the polymer matrix, although they do not exhibit higher *T_g_*. MMMs studied in this work allowed for improve selectivity, but with a negative impact on permeability. This could be caused by particle aggregation (see Figure 5 for SEM images), blocking part of the FFV but increasing tortuosity of the gases trough the membrane. Regarding the influence of the particle type and/or content, no clear effect of the particles in terms of pore size or particle size was discovered. XRD analysis shows a small decrease in interspacing of the polymer chains with no modification of the particles pattern, meaning that there is not any interaction with polymer.

Taking all the inorganic particles into consideration, BCZY shows the best improvement of selectivity with a small decrease in permeability. In addition, it also exhibits the best distribution, and consequently, it was selected for the rest of the experiments (different percentages and different polymer matrix). For polymer matrixes with lower FFV, such as Matrimid^®^ and P84^®^, there is a result in improvement of the properties by adding particles, possibly due to the creation of interface between particle and polymer chain. Therefore, MMMs with particles can be used to create interface and we will increase separation properties of slow polymers. Regarding temperature dependence, some changes were observed on activation energy of the process, the incorporation of inorganic fillers does not significantly affect the permeation mechanism determined by the polymer transport properties.

Finally, water permeability was first reported for several polyimides and MMMs of inorganic particles with polyimides, reaching relatively high values. However, the effect of the filler incorporation on the water permeation was not relevant for the polymers except for Matrimid.

## Figures and Tables

**Table 1:** Properties of the used particles. 8YSZ [43], La_2_O_3_ [44], LaWO [45], BCZY [46,47], ITQ-2 [42], and Beta [48].

Particles	8YSZ	La_2_O_3_	LaWO	BCZY	ITQ-2	Beta
Description	8% mol of Y_2_O_3_ stabilized ZrO_2_ (Tosoh)	Co-precipitation from La(NO_3_)_3_. Calcined 800 °C/5 h	La_5.4_WO_12_ (CerPoTech). Calcined 950 °C/6 h	BaCe_0.2_Zr_0.7_Y_0.1_O_3_ (CerPoTech). Calcined 950 °C/6 h	ITQ-2 zeolite. Si/Al = 50	Zeolite nano-crystalline. Si/Al = 12.5
Structure	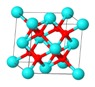	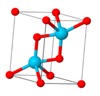	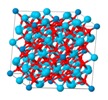	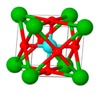	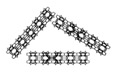	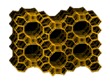
Density (g/cm^2^)	5.95	6.56	6.58	6.14	-	-
BET area (m^2^/g)	6.0	2.9	9.4	31.4	>700	>700
Size (nm)	20–80	60–100	30–120	30–100	Thin sheets (2.5 thick)	10–30
Uses	Solid electrolyte in solid oxide fuel cells (SOFC)	Ferroelectric materials and as feedstock for catalysts	Asymmetric membranes for hydrogen separation	Asymmetric membranes for hydrogen separation	Catalysis	Catalysis

**Table 2:** Thermo gravimetric analysis (TGA) and differential scanning calorimetry (DSC) results for the three different cases studied in this work.

Sample Description	*T_max loss_*	Theor. wt.%	*R_M_*wt.%	*T_g_*
6FDA-6FpDA	550 °C	0	0	311.1 °C
+10 wt.% 8YSZ	550 °C	10	9.8	300.2 °C
+10 wt.% La_2_O_3_	550 °C	10	8.1	302.4 °C
+10 wt.% LaWO	550 °C	10	8.1	290.6 °C
+10 wt.% BCZY	550 °C	10	10.0	311.2 °C
+10 wt.% ITQ-2	550 °C	10	0	294.7 °C
+10 wt.% Beta	550 °C	10	0	294.5 °C
6FDA-6FpDA	550 °C	0	0	311.1 °C
+1 wt.% BCZY	550 °C	1	0	314.2 °C
+5 wt.% BCZY	550 °C	5	4.9	312.5 °C
+10 wt.% BCZY	550 °C	10	10.0	311.2 °C
+15 wt.% BCZY	550 °C	15	16.8	307.9 °C
+20 wt.% BCZY	550 °C	20	27.2	306.3 °C
6FDA-6FpDA	550 °C	0	0	311.1 °C
+10 wt.% BCZY	550 °C	10	10.0	311.2 °C
Matrimid^®^	560 °C	0	0	320.2 °C
+10 wt.% BCZY	560 °C	10	13.3	315.7 °C
P84^®^	580 °C	0	0	322.4 °C
+10 wt.% BCZY	580 °C	10	7.2	318.2 °C

**Table 3:** CO_2_ permeability, CO_2_/CH_4_ selectivity, percentage variations of permeability and selectivity (6FDA-6FpDA with 10 wt.%fillers at 30 °C). Additionally, the activation energy for CO_2_ permeability derived from the data shown in Figure 6.

Membrane Sample Description	CO_2_ Permeability (Barrer)	CO_2_/CH_4_ Selectivity (-)	CO_2_ Permeability Variation (%)	CO_2_/CH_4_ Selectivity Variation (%)	Activation Energy (KJ/mol)
6FDA-6FpDA (Reference)	77.4	48.0	-	-	0.69
+10 wt.% 8YSZ	25.8	53.9	−67	+12	3.73
+10 wt.% La_2_O_3_	34.1	51.9	−56	+8	2.69
+10 wt.% LaWO	11.9	77.3	−85	+61	5.51
+10 wt.% BCZY	63.8	54.6	−18	+14	1.22
+10 wt.% ITQ-2	28.9	55.1	−63	+15	2.63
+10 wt.% Beta	22.7	64.9	−71	+35	4.98

**Table 4:** CO_2_ permeability, CO_2_/CH_4_ selectivity, percentage variations of permeability, and selectivity (6FDA-6FpDA with different % of BCZY at 30 °C). Additionally, the activation energy for CO_2_ permeability derived from permeability vs. temperature data is listed.

Membrane Sample Description	CO_2_ Permeability (Barrer)	CO_2_/CH_4_ Selectivity (-)	CO_2_ Permeability Variation (%)	CO_2_/CH_4_ Selectivity Variation (%)	Activation Energy (KJ/mol)
6FDA-6FpDA (Reference)	77.4	48.0	-	-	0.69
+1 wt.% BCZY	61.4	49.7	−21	+3.6	2.56
+5 wt.% BCZY	45.5	49.6	−41	+3.3	1.89
+10 wt.% BCZY	63.8	54.6	−18	+14	1.22
+15 wt.% BCZY	66.0	47.8	−15	−0.3	0.57
+20 wt.% BCZY	59.7	45.1	−23	−6.0	1.46

**Table 5:** CO_2_ permeability, CO_2_/CH_4_ selectivity, percentage variations of permeability and selectivity (membranes with different polymeric matrix at 30 °C). Additionally, activation energy is calculated.

Membrane Sample Description	CO_2_ Permeability (Barrer)	CO_2_/CH_4_ Selectivity (-)	CO_2_ Permeability Variation (%)	CO_2_/CH_4_ Selectivity Variation (%)	Activation Energy (KJ/mol)
6FDA-6FpDA (Reference)	77.4	48.0	-	-	0.69
+10 wt.% BCZY	63.8	54.6	−18	+14	1.22
Matrimid^®^	5.1	40.5	-	-	9.30
+10 wt.% BCZY	6.7	47.5	+31	+17	7.42
P84^®^	1.4	47.0	-	-	11.64
+10 wt.% BCZY	1.0	64.6	−32	+38	18.33

**Table 6:** Steam permeation at 30 °C and activation energies for all the MMMs studied in this work at 30 °C.

	H_2_O Permeability (Barrer)	H_2_O/CO_2_ Selectivity (-)	Activation Energy (KJ/mol)
6FDA-6FpDA	3875	50.06	−3.34
+10 wt.% 8YSZ	1998	77.48	−2.22
+10 wt.% La_2_O_3_	2381	69.85	−2.79
+10 wt.% LaWO	1287	108.30	−1.35
+10 wt.% BCZY	3319	52.02	−3.31
+10 wt.% ITQ-2	2015	69.70	−1.31
+10 wt.% Beta	1914	84.35	0.69
6FDA-6FpDA	3875	50.06	−3.34
+1 wt.% BCZY	3144	51.20	−2.30
+5 wt.% BCZY	2766	60.83	−2.23
+10 wt.% BCZY	3319	52.02	−3.31
+15 wt.% BCZY	3276	49.67	−2.13
+20 wt.% BCZY	3108	52.03	−2.28
6FDA-6FpDA	3875	50.06	−3.34
+10 wt.% BCZY	3319	52.02	−3.31
Matrimid^®^	1524	300.60	0.87
+10 wt.% BCZY	1835	276.02	−1.16
P84^®^	1226	875.71	2.36
+10 wt.% BCZY	821	856.40	1.73
